# Typographical Errors

**Published:** 1863-02

**Authors:** 


*Typographical Errors.*—Our readers will observe numerous typographical errors in the first sheet of this Journal. The *press work*, as printers call it, was done first, and proof reading or correction afterwards.

